# A Novel Time Domain Reflectometry (TDR) System for Water Content Estimation in Soils: Development and Application

**DOI:** 10.3390/s25041099

**Published:** 2025-02-12

**Authors:** Alessandro Comegna, Simone Di Prima, Shawcat Basel Mostafa Hassan, Antonio Coppola

**Affiliations:** 1 Department of Agricultural Forestry Food and Environmental Sciences (DAFE), University of Basilicata, 85100 Potenza, Italy; simone.diprima@unibas.it (S.D.P.); shawkat.hassan@unibas.it (S.B.M.H.); 2 Department of Chemical and Geological Sciences, University of Cagliari, 09124 Cagliari, Italy; antonio.coppola@unica.it

**Keywords:** time domain reflectometry (TDR) technique, low-cost systems, soil sensors, TDR device calibration and validation, soil water content, dielectric properties, environmental applications

## Abstract

**Highlights:**

A compact, affordable time domain reflectometry (TDR) device was designed for hydrological applications. The proposed device demonstrated reliable performance across different soil textures. Laboratory experiments validated the accuracy of the system for soil moisture estimation. The device is suitable for real-time monitoring in precision agriculture and environmental studies. The device offers a viable alternative to expensive commercial TDR systems without compromising measurement accuracy.

**What are the main findings?**

- Development of a low-cost TDR.
- Consistent and reliable performance.

**What is the implication of the main finding?**

- Possibility to build the device on one’s own.
- It makes TDR suitable for monitoring soil water status with acceptable accuracy

**Abstract:**

Nowadays, there is a particular need to estimate soil water content accurately over space and time scales in various applications. For example, precision agriculture, as well as the fields of geology, ecology, and hydrology, necessitate rapid, onsite water content measurements. The time domain reflectometry (TDR) technique is a geophysical method that allows, in a time-varying electric field, the determination of dielectric permittivity and electrical conductivity for a wide class of porous materials. Measuring the volumetric water content in soils is the most frequent application of TDR in soil science and soil hydrology. TDR has grown in popularity over the last 40 years because it is a practical and non-destructive technique that provides laboratory and field-scale measurements. However, a significant limitation of this technique is the relatively high cost of TDR devices, despite the availability of a range of commercial systems with varying prices. This paper aimed to design and implement a low-cost, compact TDR device tailored for classical hydrological applications. A series of laboratory experiments were carried out on soils of different textures to calibrate and validate the proposed measuring system. The results show that the device can be used to obtain predictions for monitoring soil water status with acceptable accuracy (*R*^2^ = 0.95).

## 1. Introduction

Time Domain Reflectometry (TDR) is a proven measurement principle for evaluating the frequency-dependent dielectric properties of various materials, including soils [1,2,3]. TDR operates by propagating and reflecting electromagnetic waves along a transmission line [4,5,6]. When a TDR instrument transmits a fast-rising voltage pulse through a coaxial cable or waveguide embedded in a medium, the wave interacts with the material’s dielectric properties. Discontinuities, such as impedance variations caused by changes in the medium, generate reflected signals that are captured and analyzed. This process yields detailed insights into the material’s physical and chemical characteristics [7,8].

The ability of TDR to assess both the dielectric permittivity and electrical conductivity of soils has made it an indispensable tool for hydrological studies [9,10,11]. Dielectric permittivity, which is strongly influenced by water content, enables the precise estimation of soil moisture.

In agricultural and environmental monitoring, TDR is widely employed to evaluate water availability, irrigation efficiency, and groundwater recharge. This versatility makes it a fundamental tool for sustainable water resource management [12,13,14,15,16]. The use of TDR offers several significant advantages over traditional soil moisture measurement techniques. One of its key benefits is the ability to perform real-time, in-situ measurements, enabling the non-destructive and continuous monitoring of soil moisture [17,18], among others. This capability is particularly valuable for studying dynamic soil processes, such as water infiltration, evapotranspiration, and drainage, across various agricultural and environmental contexts [19,20,21,22].

Another key advantage of TDR is its precision and accuracy. TDR devices can provide highly accurate soil water content measurements (typically within 2–3%) across a wide range of soil types and moisture conditions [23,24]. This precision makes TDR an indispensable tool for soil scientists, agronomists, hydrologists, and engineers who require reliable data for their research and applications [25,26,27].

In addition, the TDR technique offers a high degree of spatial and temporal resolution. TDR probes can be installed at various depths and orientations, enabling detailed profiling of soil moisture throughout the soil profile. This capability facilitates large-scale data collection and improves the accuracy of hydrological models [28,29,30]. Furthermore, TDR measurements are rapid, allowing the monitoring of soil moisture fluctuations over short time scales. This enables valuable insights into transient water movement, root zone behavior, and other critical soil–water interactions [31,32].

Although TDR has revolutionized soil hydrology studies, the high cost of commercial TDR systems has limited its widespread adoption, particularly in resource-constrained settings. Standard systems are often prohibitively expensive, making them inaccessible to small-scale farmers, community-based environmental monitoring programs, and educational institutions, especially in developing countries.

The growing demand for affordable alternatives has driven innovation in designing low-cost TDR systems. Such systems capitalize on advancements in electronics, microcontrollers, and open-source technologies to substantially reduce costs without compromising performance. For example, Arduino-based TDR systems have emerged as a practical and cost-effective solution for researchers and practitioners operating on limited budgets, even though systems strictly based on this technology may exhibit low accuracy due to low temporal resolution, the lack of advanced signal conditioning circuits (TDR signals require specialized hardware for generating and detecting high-frequency pulses), noise, and interference [15,33,34,35].

Moreover, the integration of low-cost TDR systems with wireless communication technologies, remote sensing, and IoT platforms is transforming soil hydrology into a data-rich discipline. Real-time data collected from multiple locations can be aggregated and analyzed remotely, offering actionable insights for policymakers, water managers, and farmers [36,37,38].

After briefly recalling the main theoretical principles of the TDR technique, this paper provides details on designing and implementing the proposed TDR device. In summary of the research, several laboratory-controlled tests were carried out to calibrate and validate the proposed measuring system, using four selected soils with different textures.

## 2. TDR Technique: Theoretical Background and Measurement Principles

**Figure 1:**
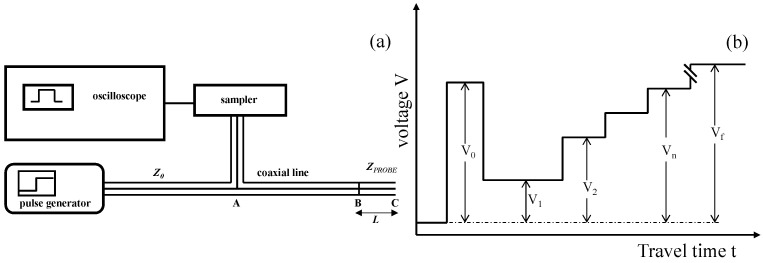
(**a**) Time Domain Reflectometry (TDR) hardware setup; (**b**) voltage versus travel time for an idealized TDR waveform, highlighting distinctive signal characteristics resulting from multiple reflections (adapted from [6]).

A schematic representation of a TDR measurement system is shown in Figure 1a. The system consists of a TDR device, comprising an oscilloscope, a pulse generator, a sampling unit, and a TDR probe of length *L*. The probe is connected to the TDR device via a coaxial cable with a nominal impedance of Z_0_ = 50 Ω. The entire system can be controlled through a computer.

The principle of the TDR technique is based on measuring the propagation velocity of a step voltage pulse along a TDR probe embedded in the soil [39]. The probe is installed such that its metal rods are fully surrounded by the soil material. The TDR device generates short electromagnetic pulses, typically in the frequency range between 20 kHz and 1.5 GHz, which propagate along the coaxial cable and then along the rods of the TDR probe [40].

As the electromagnetic energy travels, part of it is reflected at points where abrupt changes in relative permittivity occur. Initially, the signal reaches point A, where the voltage step *V_0_* is detected and displayed on the oscilloscope (Figure 1b). The signal then enters the coaxial line connecting the probe to the TDR equipment. At the head of the TDR probe (point B, corresponding to the cable–probe interface), the signal encounters a change in impedance (*Z*_PROBE_) at the base of the probe [6]. This impedance mismatch causes a portion of the electromagnetic energy to reflect back toward the source (i.e., the generator). The reflected energy may interfere in-phase or out-of-phase with the newly generated signal [25].

The remaining energy propagates through the soil along the metal rods, which act as a waveguide, until it reaches point C. At this point, the signal is fully reflected due to the open-ended design of the probe, which presents an infinite impedance [23,25,41,42,43].

As the signal travels, the reflections cause variations in the measured voltages (i.e., *V*_1_, *V*_2_,…,*V_n_*, …, *V_f_*). By analyzing the signal response, it is possible to determine the dielectric properties of the material through which the electromagnetic pulse propagates.

**Figure 2:**
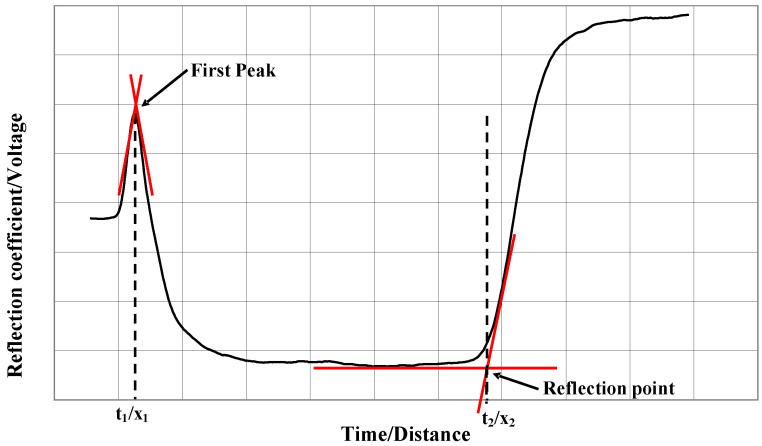
Example of a TDR output waveform, showing (i) the first peak, (ii) the reflection point, and (iii) tangent lines required for the determination of these two points.

In particular, determining the travel time of the signal along the TDR rods through the soil requires identification of two significant reflections: the first occurs at the head of the TDR probe (commonly referred to as the first peak) and the second occurs at the end of the rods (commonly referred to as the reflection point). These key reflections are illustrated in Figure 2.

From these two characteristic points, the two-way travel time of the electromagnetic signal through the soil can be determined. Using this information, the composite or bulk dielectric permittivity of the material surrounding the probe rods can be calculated as:

(1) $$\varepsilon_{b}={\left(\frac{ct}{2L}\right)}^{2}$$

where *c* (ms^−1^) is the velocity of an electromagnetic wave in free space, *L* is the probe length, and *t* is the effective time (i.e., travel time) the TDR signal takes to travel along the probe rods.

The travel time *t* can be determined using the tangent line method [44], which involves calculating the first derivative of the TDR signal (not shown in Figure 2). The reference points for t calculation are identified by selecting the highest derivative values, corresponding to the impedance changes at the probe head and at the end of the rods.

The soil volumetric water content (θ) can be determined from the measured permittivity using Topp’s equation [23], as follows:

(2) $$\theta =-5.3\cdot 10^{-2}+2.92\cdot 10^{-2}\varepsilon_{b}-5.5\cdot 10^{-4}\varepsilon_{b}^{2}+4.3\cdot 10^{-6}\;\varepsilon_{b}^{3}$$

which is a third-order polynomial equation.

## 3. Materials and Methods

### 3.1. Hardware Description

In this project, we developed a low-cost TDR system, referred to as PoKetTDR (PKTDR). For proper operation, the PKTDR device must be connected to a digital storage oscilloscope (DSO) and a PC. The PC provides 5-volt power and facilitates the visualization, acquisition, and storage of TDR signals. The selected DSO is the Hantek 6254BD, a four-channel oscilloscope with a total bandwidth of 250 MHz, a real-time sampling rate of 1 GSa/s, and a data storage capacity of 4096 data points.

The PKTDR system incorporates a pulse generator with a rise time of approximately 3 ns, resulting in a maximum effective frequency of nearly 120 MHz.

**Figure 3:**
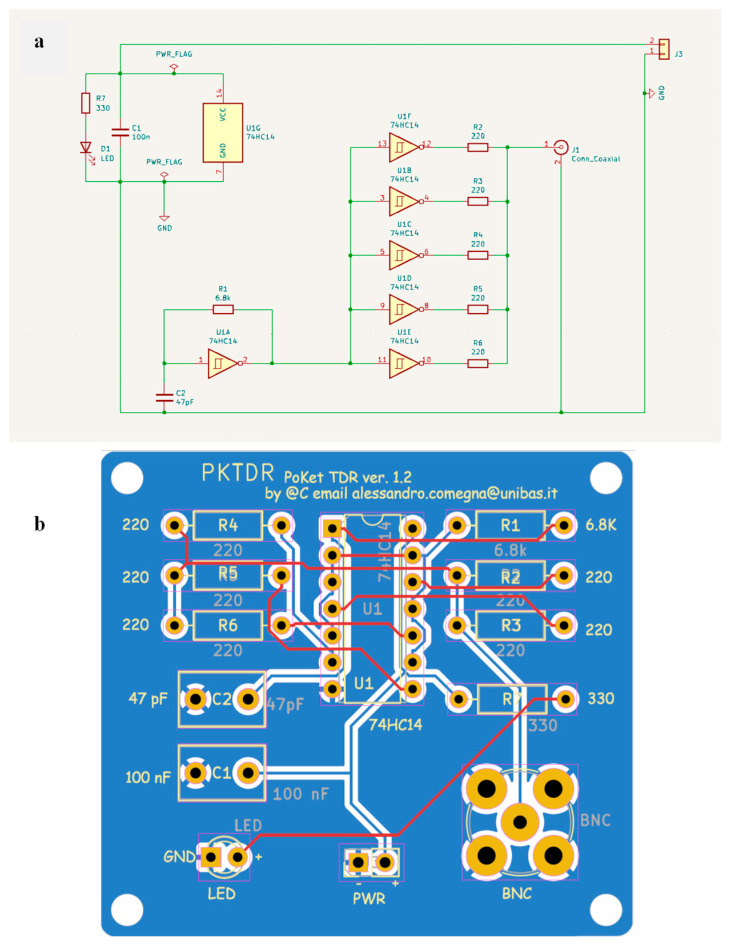
(**a**) Electrical circuit diagram of the PKTDR device and (**b**) the printed circuit board (PCB) layout generated using KiCad software (version 7.0).

Figure 3a,b present the wiring diagram and printed circuit board (PCB) layout of the proposed TDR device.

**Figure 4:**
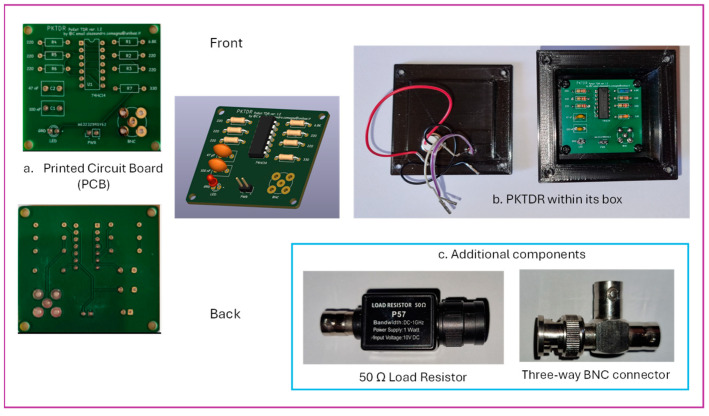
(**a**) The printed circuit board (PCB) of the PKTDR device, (**b**) PKTDR housed within its PLA enclosure, and (**c**) the additional components needed to complete the assembly.

Figure 4a–c display the hardware components required for assembling the PKTDR device. The printed circuit board (PCB) shown in Figure 4a was designed using KiCad software (accessed on 20 December 2024 https://www.kicad.org/), resulting in a board size of 46 mm × 50.5 mm. Figure 4b illustrates the PKTDR housed within a 3D-printed enclosure, whereas Figure 4c presents the additional components required for assembly.

Table 1 provides the Bill of Materials (BoMs) required for assembling the TDR system. Additional details regarding the assembly of the PKTDR device, its implementation costs, and all the required building materials are provided in Appendix A.

**Figure 5:**
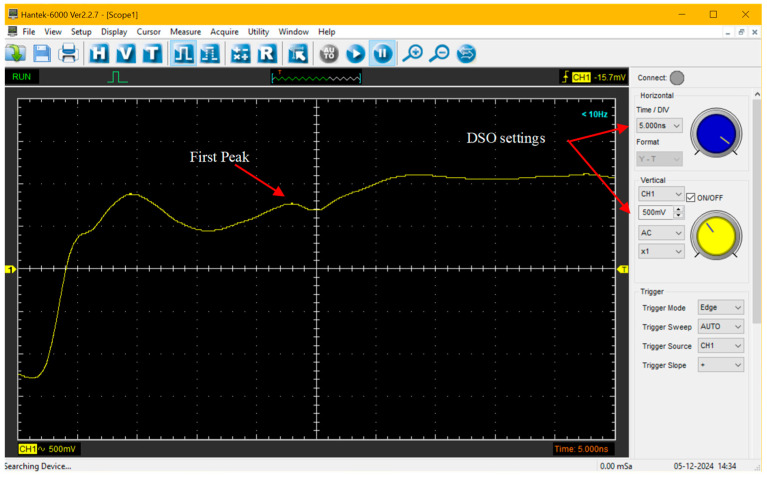
Example of a TDR signal acquired using PKTDR with the Hantek 6254BD DSO.

Figure 5 illustrates an example of a TDR signal acquired using PKTDR + DSO as acquired from the Hantek 6000 software (version 2.2.7) included with the Hantek 6254BD. The DSO parameters for accurate TDR signal acquisition are displayed on the right. Specifically, the Time/div setting was fixed at 5 ns and the vertical DSO scale (i.e., voltage) was set to 500 mV.

Finally, we would like to emphasize that, in its current stage of development, the PKTDR is limited to measuring bulk dielectric permittivity. Future studies will aim to enhance its capabilities, focusing on the potential to also estimate soil bulk electrical conductivity.

### 3.2. Soil Characterization and Experimental Setup

A series of laboratory experiments were conducted to calibrate and validate the PKTDR apparatus, using repacked soil samples collected from the Ap horizon of four soil sites. We selected a sandy loam soil (hereinafter referred to as SALO), a silty loam soil (SILO), a loam soil (LOAM), and a sand soil (SAND), classified according to the IUSS Working Group WRB [45]. Table 2 presents the main physical properties of the selected soils.

Soil texture was determined using the method proposed by [46]. Soil bulk density (*ρ_b_*) and pH were measured using the methods described in [47,48], respectively.

The experimental setup included a PKTDR device (comprising a DSO and TDR unit), a commercial TDR 100 apparatus (Campbell Scientific, Logan, UT, USA), and a three-wire TDR probe with 14.5 cm long guides, connected to the tester via a 2 m long RG58 coaxial cable. The TDR signals were collected using a PC-based data acquisition system.

MATLAB code, version 2024a, (refer to the User’s Guide of the MATPKTDR code in the available Supplementary Material) was developed for post-processing the acquired TDR waveforms. In particular, the MATPKTDR code was specifically developed for the PKTDR device connected to the Hantek 6254BD oscilloscope. The code is also compatible with commonly used commercial TDR devices, such as the Tektronix 1500 series and the TDR100. The MATPKTDR code requires the acquired TDR signal to be provided as an ASCII file and a set of input parameters to be configured for proper operation.

**Figure 6:**
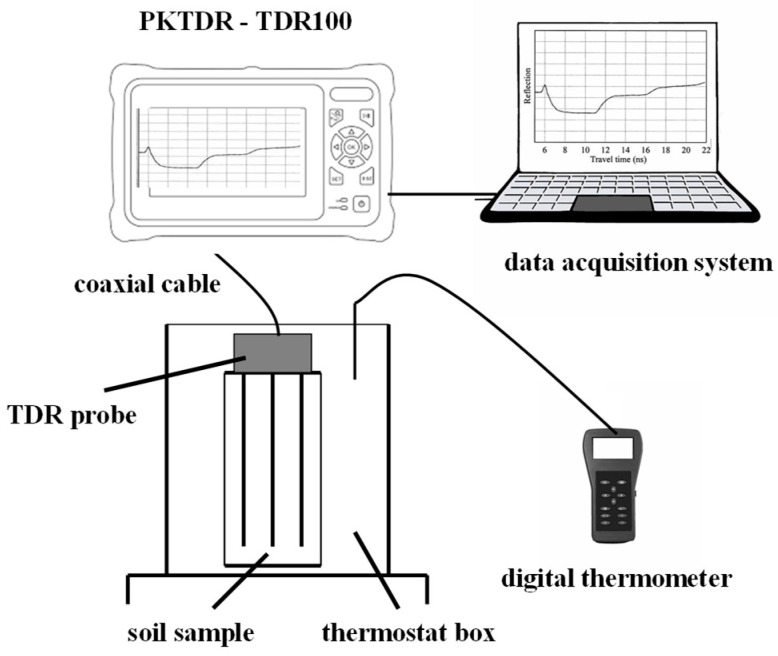
Laboratory apparatus for calibration and validation of the PKTDR device (adapted from [4]).

Figure 6 illustrates the dielectric measurement system used in the experiments.

During the experiments, the PKTDR system was tested to estimate the volumetric water content of soil samples across a range of θ values from 0 to 0.40. In detail, for each soil, samples were initially oven-dried at 105 °C and sieved at 2 mm. Known amounts of soil and water were then mixed, shaken, and stored in sealed plastic bags for 24 h to ensure uniform water distribution. Following this preliminary phase, the soil samples were placed in cylindrical PVC containers (9.5 cm in diameter and 15 cm in height). During packing, the soil was carefully compacted in multiple steps to achieve the desired bulk density, which was kept constant throughout all tests. A nylon gauze was used to secure the bottom of each sample and prevent soil loss.

After repacking, θ measurements were taken at specific water content levels (θ = 0, 0.10, 0.15, 0.20, 0.30, and 0.40) using the PKTDR device. For comparison, measurements were also performed using the TDR 100 device. Additionally, as a standard reference, θ values were determined using the thermo-gravimetric method [24].

To eliminate potential discrepancies due to probe geometry, all measurements were conducted with the same TDR probe. Since TDR readings can be influenced by temperature [44], soil samples were tested within a temperature range of 20–30 °C, with 1 °C increments. In all, 300 measurements (6 soil samples × 10 temperature levels × 5 replicates for each soil) were collected. Each measurement is the average of 10 replicates. Separate datasets were prepared for each soil, as performed during the calibration phase, to be used for model validation.

### 3.3. Statistical Indices for Sensor Performance Evaluation

The performance of the proposed TDR apparatus was assessed using different statistical indices: (i) the mean bias error (*MBE*), (ii) the maximum absolute percentage error (*ME*), and (iii) the mean absolute percentage error (*MAE*), computed based on the following formulas [49,50]:

(3) $$MBE=\frac{\sum_{i=1}^{N}\left(E_{i}-O_{i}\right)}{N}$$

(4) $$ME\left(\%\right)=max\left|E_{i}-O_{i}\right|\cdot 100$$

(5) $$MAE\left(\%\right)=\frac{\left|E_{i}-O_{i}\right|}{N}\cdot 100$$

where $O_{i}$ is the true value (i.e., obtained using the thermo-gravimetric method), $E_{i}$ is the prediction (i.e., TDR estimation), $\bar{O}$ is the mean of the observed data, and *N* is the number of observations.

## 4. Results and Discussion

**Figure 7:**
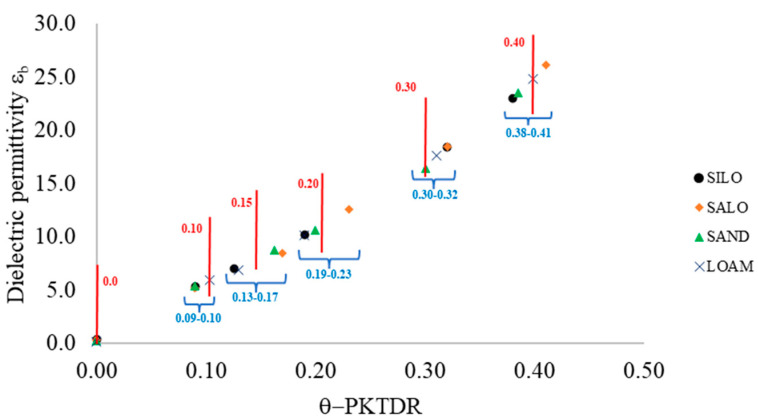
Bulk dielectric permittivity (ε_b_) values measured using the PKTDR device plotted against the estimated θ values, calculated using Equation (2) for the four investigated soils. The red lines and red text represent θ values determined using the thermo-gravimetric method. The graphic also highlights the range of θ variability among the soils in blue text.

Figure 7 shows the results of the experiments conducted on the four selected soils. For each soil, the figure presents the estimated bulk dielectric permittivity values, obtained using the PKTDR device, plotted against the θ values calculated using Equation (2). The figure also includes the expected θ values (i.e., those obtained using the thermo-gravimetric method, represented by vertical red lines) and the range of variability of the estimated θ values across the soils (indicated by curly brackets and blue text).

In the observed θ domain, $\varepsilon_{b}$ values rise with increasing volumetric water content, in accordance with the theoretical principles of the TDR technique. For a given expected θ value, variations in the observed bulk dielectric permittivity values (and consequently in θ) can primarily be attributed to mineralogical differences among the soils, as well as to experimental procedures [51,52,53].

**Figure 8:**
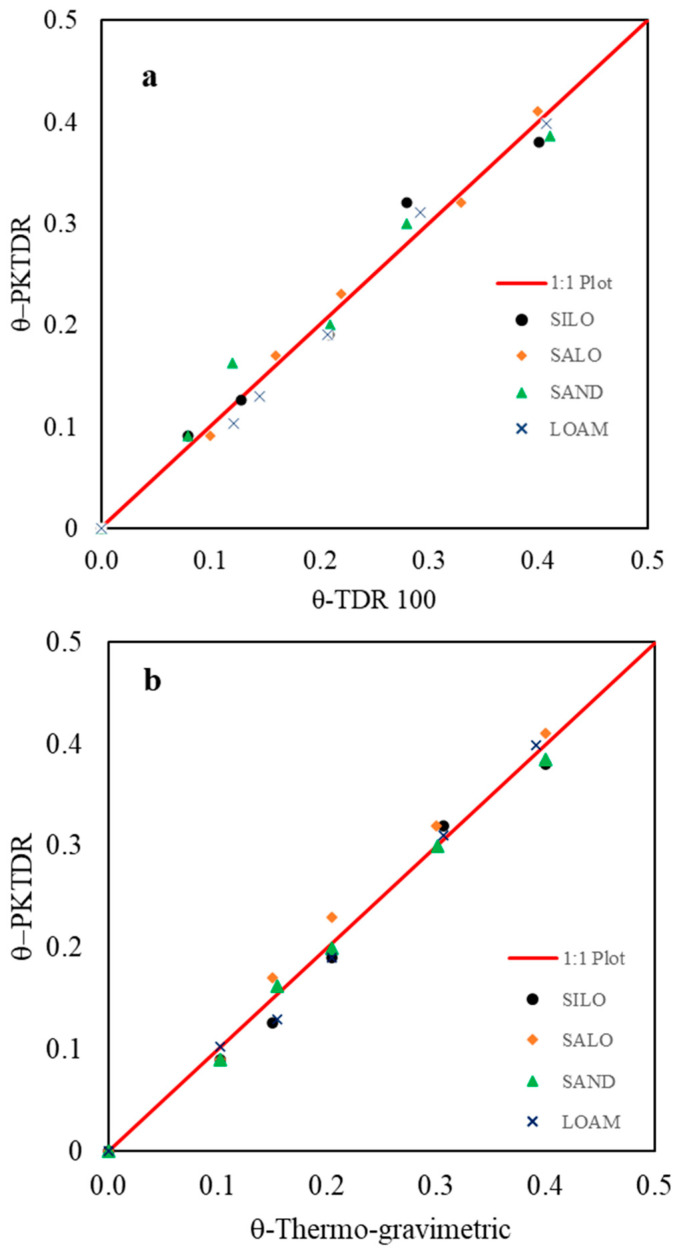
(**a**) Comparison of θ values estimated using the PKTDR device and the commercial TDR100 device and (**b**) θ-PKTDR estimated values vs. known θ values, with reference to the four investigated soils.

In Figure 8a, the θ values derived from the PKTDR device are compared with those obtained using the commercial TDR100 device. Among the soils, the dielectric responses of the two TDR devices are generally similar and closely align with the 1:1 line, indicating that the PKTDR provides performance comparable to that of the TDR100.

Finally, Figure 8b presents a comparison between the θ values estimated using the PKTDR device and those obtained using the thermo-gravimetric method. The 1:1 trend line has a coefficient of determination of *R*^2^ = 0.94, indicating that the proposed TDR device demonstrates high accuracy in predicting θ values compared to the standard method.

For a comprehensive evaluation, Table 3 summarizes the statistical indices (*MBE*, *ME*, and *MAE*) that assess the goodness of fit between measured (thermo-gravimetric method) and estimated θ values (PKTDR and TDR100 devices). The table also reports the standard deviation of the ε_b_ values obtained across different soil temperatures.

In general, both devices exhibited low values for the selected statistical indices across the different soils, indicating a good response of the two compared TDR systems. The MAE values for PKTDR are consistently below 2%, confirming its high accuracy for all tested soil types. The maximum error is also minimal, ranging from 0.69 (for LOAM soil) to 2.52 (for SALO soil). Furthermore, analysis of the standard deviation of ε_b_ values (σ_ε_) across different temperature ranges shows that PKTDR maintains good thermal stability, with values differing only slightly among soils. These results underscore PKTDR’s robustness and precision under varied environmental conditions.

## 5. Conclusions

In the present study, based on several full factorial laboratory experiments, we assessed the performance of a low-cost TDR device across four soils with different textural characteristics.

This study demonstrated the accuracy and robustness of the PKTDR device, showing a strong correlation between measured and actual soil water contents, from saturated to dry soil conditions. The economic accessibility of the proposed TDR device should encourage widespread adoption, particularly in resource-limited settings, such as those found in developing countries and among small-scale farming communities, as well as for extensive (i.e., open-field) applications.

Emerging trends in TDR research include the integration of TDR systems with remote sensing technologies, such as unmanned aerial vehicles (UAVs) and satellites, to enable the large-scale monitoring of soil moisture across vast landscapes. This integration has the potential to revolutionize agricultural and environmental monitoring by providing real-time, high-resolution data over large spatial areas. For these reasons, to extend the potential of the proposed TDR system, future perspectives will focus on the possibility of integrating the proposed system with IoT technologies. Finally, in the upcoming phases, extensive field-scale tests will be conducted to assess sensor performance under open-field conditions.

## Figures and Tables

**Table 1:** List of electronic components (BoMs) required for assembling the PKTDR system.

Footprint Assignment	Designator	Quantity	Footprint Specification (Kikad)	Mounting Type
C1	Capacitor 100 nF	1	THT:C_Disc_D8.0 mm_W5.0 mm_P5.00 mm	THT *
C2	Capacitor 47 pF	1	THT:C_Disc_D8.0 mm_W5.0 mm_P5.00 mm	THT
D1	Led (3 mm)	1	LED_THT:LED_D3.0 mm	THT
J1	BNC Connector	1	BNC_TEConnectivity_1478204_Vertical	THT
J3	Connector	1	PinHeader_2.54 mm:PinHeader_1 x02_P2.54 mm_Vertical	THT
R1	Resistor 6.8 kΩ	1	THT:R_Axial_DIN0207_L6.3 mm_D2.5 mm_P10.16 mm_Horizontal	THT
R2-R6	Resistor 220 Ω	5	THT:R_Axial_DIN0207_L6.3 mm_D2.5 mm_P10.16 mm_Horizontal	THT
U1	Inverter 74 HC14	1	Package_DIP:DIP-14_W7.62 mm	THT

* THT: Through-Hole Technology Mounting.

**Table 2:** Main physical and chemical properties of the four selected soils.

Soil ID	Depth (cm)	Soil Texture and Classification (USDA)				*ρ_b_* (g/cm^3^)	*pH*
		Texture	Sand (%)	Silt (%)	Clay (%)		
SALO	0–20	sandy loam	57.43	31.95	10.62	1.02	7.7
SILO	0–20	silty loam	15	72.7	11.6	1.02	8.4
LOAM	0–20	loam	27.5	46.1	26.4	1.02	7.2
SAND	0–20	sand	98	1.5	0.5	1.02	7.9

**Table 3:** The mean bias error (*MBE*), maximum absolute percentage error (*ME*), and mean absolute percentage error (*MAE*) statistical indices and the standard deviation σ_ε_ of bulk dielectric permittivity (determined in the 20–30 °C range), referring to the PKTDR and TDR100 measured and calculated (thermo-gravimetric method) θ values, with reference to the four selected soils.

Soil	PKTDR	TDR100	PKTDR	TDR100	PKTDR	TDR100	PKTDR	TDR100
	*MBE*		*ME* (%)		*MAE* (%)		σ_ε_	
SALO	0.0104	0.0087	2.52	3.0	1.45	0.95	0.0158	0.0140
SAND	−0.0043	−0.0103	0.78	1.09	0.69	1.57	0.0131	0.0126
LOAM	−0.0048	0.0023	0.69	1.91	0.82	1.03	0.0149	0.0139
SILO	−0.0099	−0.0112	1.28	0.40	1.42	1.27	0.0164	0.0150

**Figure A1:**
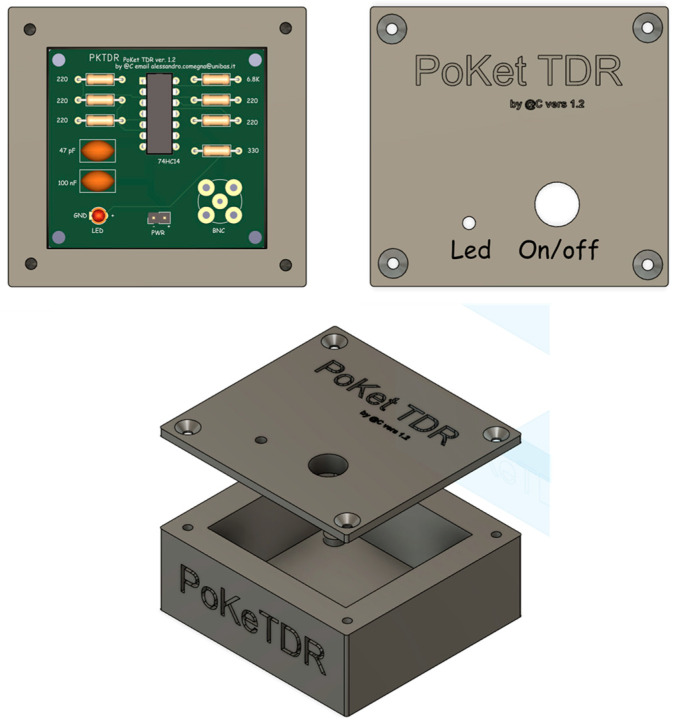
The PKTDR prototype.

**Figure A2:**
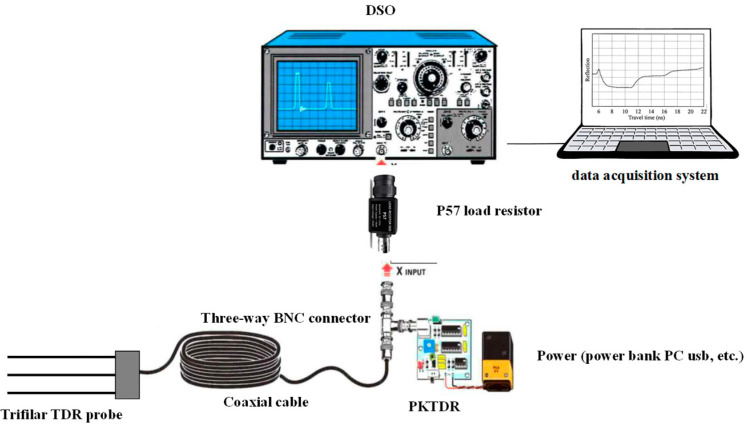
Scheme of the required components and connections for the PKTDR system.

## Data Availability

The original contributions presented in this study are included in the article/Supplementary Material. Further inquiries can be directed to the corresponding author.

## Supplementary Materials

The following supporting information can be downloaded at: https://www.mdpi.com/article/10.3390/s25041099/s1.

## Appendix A. Supplementary Information

This section provides supplementary information essential for assembling the PKTDR device. All the essential files, including PCB fabrication files, 3D printing files, etc., for building the proposed device are available as supplementary material accompanying this paper. The supplementary material folder is organized as follows:

### Appendix A.1. Board Fabrication Files Folder

(a) The Gerber files (PKTDR.kicad_pcb.zip);
(b) The Centroid files (PKTDR-bottom-pos.cvs, PKTDR-top-pos.csv);
(c) The Bill of Materials (BoMs: PKTDR_BoM.xls).

All the electronic components can be acquired in different electronics stores (e.g., JLCPCB, Mouser, Digikey, RSOnline, etc.).

### Appendix A.2. MATPKTDR Software Folder

(a) mainMATPKTDR: the primary MATLAB file (the source code) dedicated to calculating bulk dielectric permittivity and volumetric water content (specifically developed for the PKTDR + Hantek 6254BD system);
(b) User guide: a practical guide for using the MATPKTDR Code;
(c) General Trace Information Library folder: a repository with templates for input files (useful when using commercial TDR devices or PKTDR);
(d) TDRExample file: a .csv file containing an example of the TDR probe (to be used with the General Trace Information 4096 file);
(e) The TDROUT file: a .txt file that records the time and date of the TDR analysis along with the estimated εb and θ values;
(f) The Plot file: a .png file, which contains the graphical output of the TDR trace analysis.

### Appendix A.3. 3D Printing Materials Folder

(a) PKTDR Box Folder: .stl files for printing the external box for the PKTDR device.

The 3D printing model (Figure A1) was developed using Fusion 360 software (accessed on 20 December 2024 https://www.autodesk.com).

For building the PKTDR device, it is advisable to utilize a specialized electronics manufacturing service capable of producing low-cost printed circuit boards (PCBs). This option requires the Gerber and BoM files (refer to the Board Fabrication Files folder in the Supplementary Material). Fabrication costs are around $5 USD for printing 10 PCBs, excluding shipping. The estimated cost of electronic materials is approximately $3–5 USD.

### Appendix A.4. How to Assemble the PKTDR System

The PKTDR system requires additional components to function properly. In addition to a DSO, the system needs a load resistor (e.g., the P57 model) to ensure a stable 50-ohm output signal. A three-way BNC connector is also necessary to facilitate connections between the PKTDR, the TDR probe, and the DSO. The P57 load resistor should be placed between this connection and the DSO. Both the DSO and the PKTDR need to be powered (at 5 volts) and connected to a PC. For this, USB-C cables are required. Figure A2 shows a scheme of the required components and connections.
