# Supplementary material for: A Novel Time Domain Reflectometry (TDR) System for Water Content Estimation in Soils: Development and Application
Source: Sensors (Basel). 2025 Feb 12;25(4):1099. doi: 10.3390/s25041099 (PMC11859294; doi:10.3390/s25041099)
Supplement: Supplementary file 1 [file sensors-25-01099-s001.zip › 02 MATPKTDR Software folder/MATPKTDR/User Guide/MATPKTDR User Guide ver 1.0.pdf]

Users Guide for the MATPKTDR code

A MATLAB-based program developed to measure the volumetric water content (θ) of soils by means of the Time Domain Reflectometry (TDR) technique.

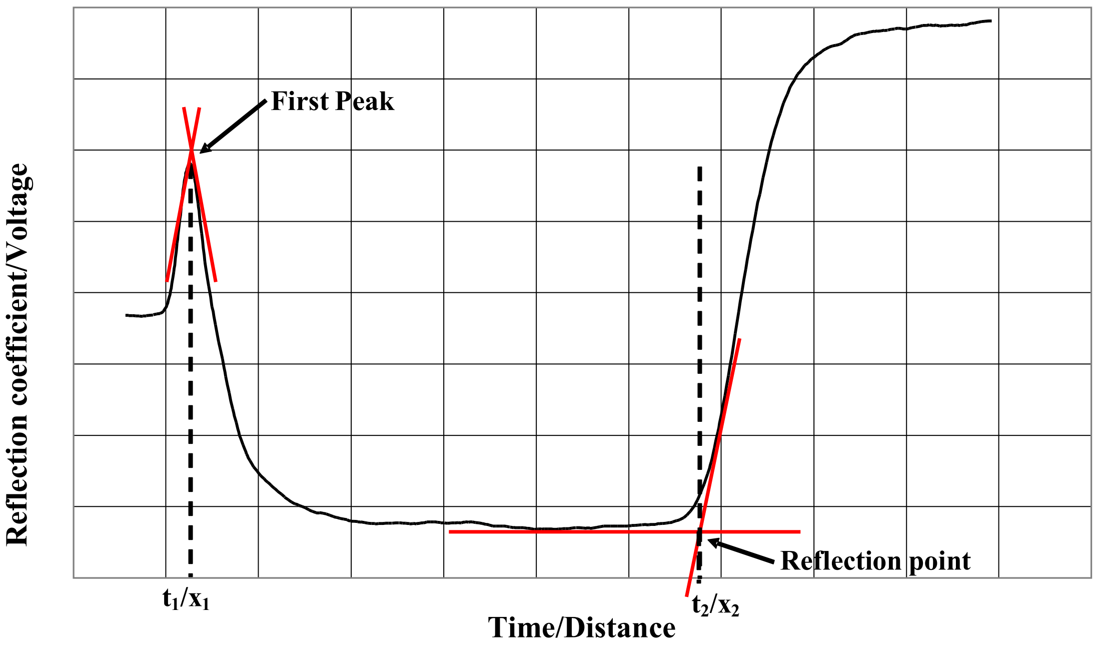

by Prof. *Alessandro COMEGNA*

Department of Agricultural, Forest, Food and Environmental Sciences (DAFE), University of Basilicata, Potenza, Italy alessandro.comegna@unibas.it

Version 1.0 (*Winter 2025*)

Introduction

The MATPKTDR code provides an easy-to-use algorithm for analyzing TDR signals with accuracy and efficiency. Specifically developed for the PKTDR device connected to the Hantek 6254BD oscilloscope, which captures signals of up to 4096 points, the code is also compatible with commonly used commercial TDR devices, such as the Tektronix 1500 series and the TDR100.

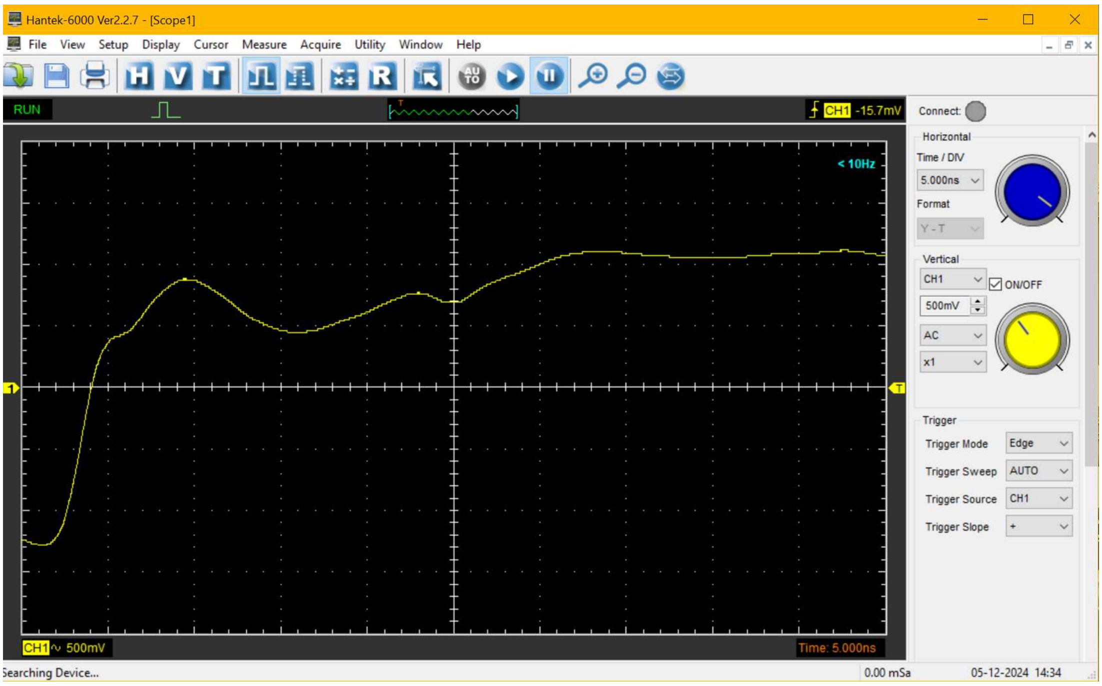

Figure 1 TDR waveform acquired via PKTDR connected to the Hantek 6254BD.

The basic principles used by the MATPKTDR code to determine the bulk dielectric permittivity (ε) from a TDR waveform are as follows. The time that an electromagnetic wave travels from the TDR device, through cables with fixed dielectric properties, to the probe head and back again is referred to as the *first reflection* or *first peak* of the TDR signal. In the current MATLAB code, this point is set during an initial calibration phase by the user. The round-trip travel time along the length (L) of the TDR probe is determined by locating the *reflection point*, which follows the first peak. Once the first peak and the reflection point are determined, the travel distance along the probe (i.e. the time difference between the first peak and the reflection point: Δt) determines the bulk dielectric permittivity (ε_b) using the following equation:

$$\varepsilon_b = \left(\frac{c\Delta t}{2L}\right)^2 \quad (1)$$

where c is the speed of light in the vacuum.

The ε_b can be then converted into the volumetric water content (θ) using Topp's equation (1980):

$$\theta = -5.3x10^{-2} + 2.92x10^{-2}\varepsilon_b\text{-}5.5\,x10^{-4}\varepsilon_b^2 + 4.3x10^{-6}\,\varepsilon_b^3 \quad (2)$$

1 Getting Started with MATPKTDR

The MATPKTDR code operates within the MATLAB environment. The following steps outline the process for setting up accurate TDR signal analysis to estimate volumetric water content (θ):

The user must first upload the MATPKTDR folder to their computer. The main folder includes the following essential files:

a) mainMATPKTDR: the primary MATLAB file dedicated to calculating bulk dielectric permittivity and volumetric water content;

b) General Trace Information file: a text file containing the input parameters required for accurate TDR signal analysis;

c) an ASCII file (.csv) containing the acquired TDR signal. An example file (TDRExample.csv) is included in the folder;

d) an output file (TDROUT): a .txt file that records the time and date of the TDR analysis along with the estimated ε_b and θ values.

e) an output file (Plots): a .png file that records the different available plots as an external image.

The main folder also contains two subfolders: General Trace Information Library: A repository with templates for input files, useful when using PKTDR or commercial TDR devices, and the User Guide which contains the present user guide.

Figure 2 illustrates the main menu of the code (i.e., the mainMATPKTDR file) after initialization.

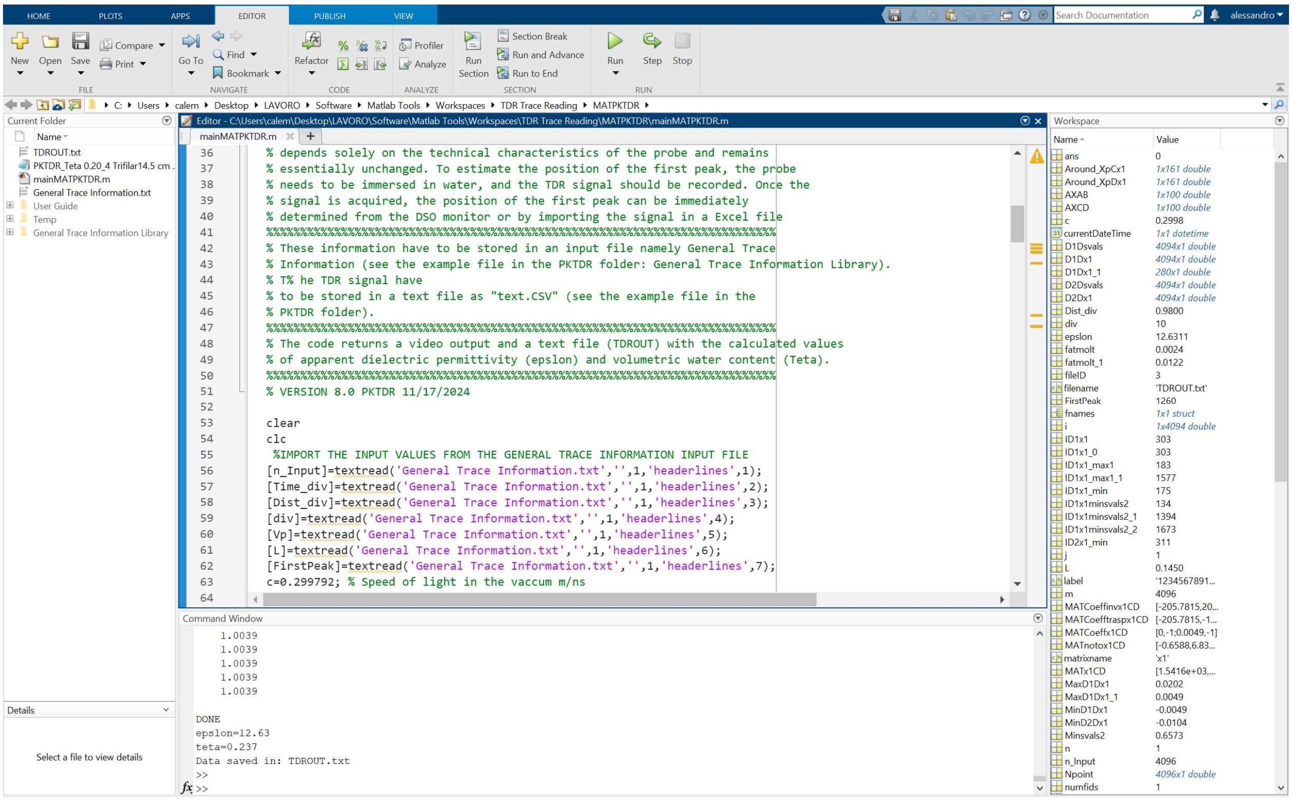

Figure 2 The main menu of the MATPKTDR code displayed after user initialization.

1.1 the main MATPKTDR file

After opening the mainMATPKTDR file, the user simply needs to execute the code by typing the *mainMATPKTDR* command in the MATLAB *Command Window*. Within a few seconds, the code analyzes the TDR signal and provides the dielectric permittivity (ε_b) and volumetric water content (θ) as a video output in the *Command Window* (Figure 3). Additionally, the results, including the output TDR information, are saved in a text file (TDROUT.txt) for the user's reference.

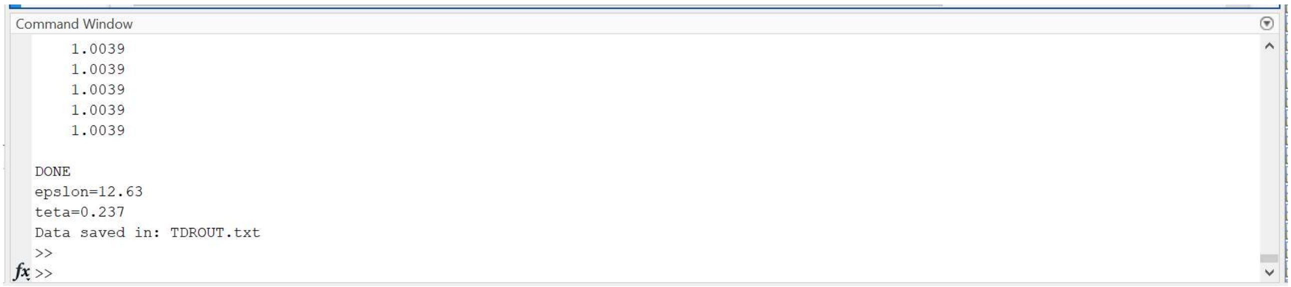

Figure 3 Video output generated by the MATPKTDR code.

1.2 the General Trace Information file

This file is essential for the correct implementation of the code. It contains all the required input parameters for TDR signal analysis, as follows:

-1- Number of Input Points (n_Input): The total number of points in the signal.

-2- Time/div (Time_div): Time per division, obtained from the DSO or TDR device.

-3- Distance/div (Dist_div): Distance per division, obtained from the DSO or TDR device.

Note: For DSOs (Digital Storage Oscilloscopes) where only Time/div is available, use the following equation to calculate Dist/div:

$$\frac{Dist}{div} = \mathrm{V_p} * c * \frac{Time}{div} \tag{3}$$

where c is the speed of light in the vacuum (0.299792 m/ns), and V_p is the relative velocity of propagation (see point 5 below).

-4- Divisions (div): The number of divisions on the TDR or DSO monitor (usually 10).

-5- Propagation Velocity (V_p): This term must be determined from the DSO and is typically set to 0.66 (i.e., 66% of *c*).

Note: when using a DSO, the V_p term must be calculated using the following equation:

$$V_p = 2 * L_{cable}/c * t \tag{4}$$

where L_{cable} is cable length and t is the time between the signal origin and the cable reflection, as observed on the DSO monitor.

In the present code, ε_b and therefore θ can also be obtained even if V_p is unknown. In this case, set V_p=0 in the General Trace Information input file.

-6- Probe Length (L): The physical length of the TDR probe, in meters.

-7- First Peak Position (FirstPeak): The abscissa of the first peak readable from the DSO display.

Figure 4 illustrates an example of the General Trace Information input file used for PKTDR waveforms.

```
General Trace Information - Blocco note di Windows
File  Modifica  Formato  Visualizza  ?
"INPUT FILE DATA second row: number of points, third row: Time/div, 4th row: Dist/div, 5th row: Vp 6th row: probe lenght 7th row: probe offset 8th row: FirstPeak"
4096
5
0.98
10
0
0.145
1260
```

Figure 4 Example of a General Trace Information file for PKTDR waveforms.

As shown in the file, each input parameter is listed on a separate line. In the example provided, 4096 refers to the number of points in the TDR-acquired waveform; 5 is the Time/div term (i.e., 5 ns per division); 0.98 is the Distance/divisions term (the user may already have this value, or if using a DSO, it should be calculated using equation 3); 10 is the number of divisions (this information can be easily determined from the TDR/DSO device); the sixth line refers to V_p, which should be set to 0 if the user does not have this information (the MATPKTDR code, as mentioned above, can also calculate ε_b and hence θ); 0.145 refers to the probe length, and finally, 1260 is the position of the first peak (as defined by the user).

It is important to note that the location of the first peak in the TDR signal depends on the technical characteristics of the probe and remains essentially unchanged (although slight variations may be observed with temperature). To estimate the position of the first peak, the probe must be immersed in water, and the TDR signal should be recorded. Once the signal is acquired, the position of the first peak can be easily determined either from the DSO monitor or by importing the signal, for example, into a spreadsheet (for e.g., Excel) file. At this point, determining the location of the first peak is straightforward (see Figure 5).

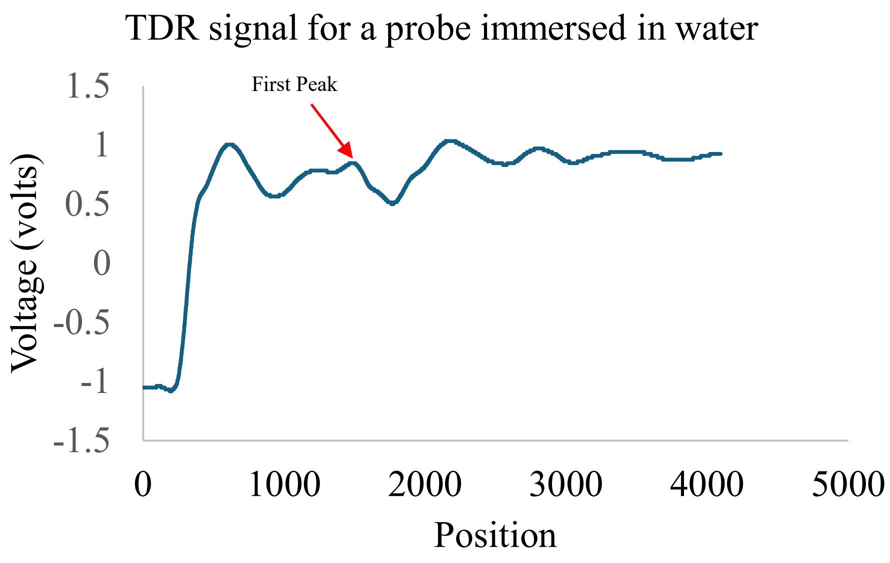

Figure 5 Example of a TDR signal for a 14.5 cm trifilar probe immersed in water.

Note: When using the General Trace Information file from the General Trace Information Library folder, the file must be renamed before use. For example, if the user needs to use the file General Trace Information 4096, it should be renamed to 'General Trace Information Library' before launching the main code.

1.3 TDR input file

The TDR input file is a .csv file containing the acquired TDR waveform. Figure 6 provides an example of such a file, which corresponds to a 4096-point TDR signal acquired by coupling the PKTDR with the Hantek 6254BD oscilloscope model. Figure 7 presents the graphical representation of the acquired TDR waveform as captured using the PKTDR.

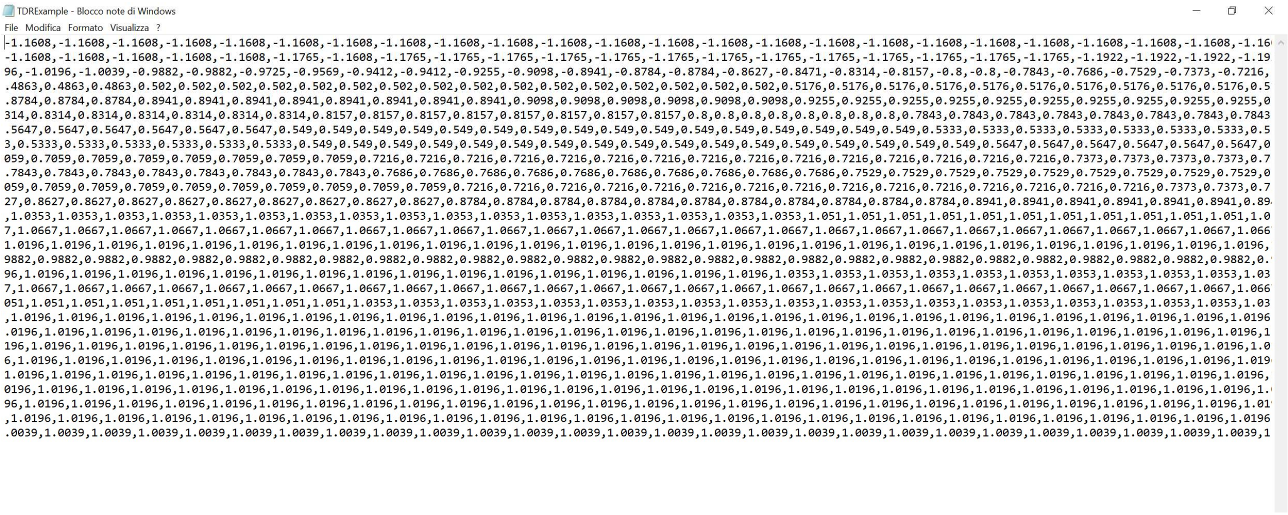

Figure 6 Example of a TDR acquired waveform file.

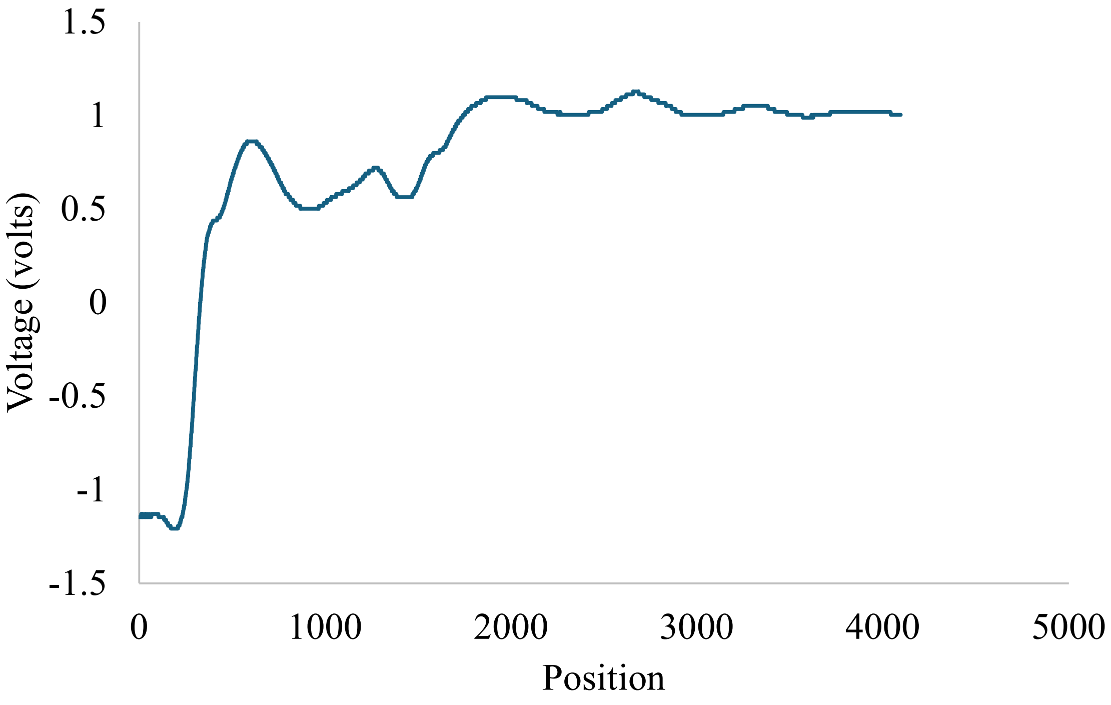

Figure 7 Example of a 4096-point TDR signal acquired via PKTDR.

Note: how to build a .csv file

The input file containing the acquired TDR signal must be a .csv file organized as shown in figure 6 (i.e., values separated by commas). To create this file, the user must import the output file generated by the DSO (as is well known, DSO management software allows observed signals to be stored in external files) into a spreadsheet application such as Microsoft Excel.

Once imported, the data are typically arranged in a single column (figure 8). To generate the correct .csv file, the user needs to copy the TDR signal, paste it as transposed data (figure 9), and save it in **CSV (Comma-Separated Values)** format.

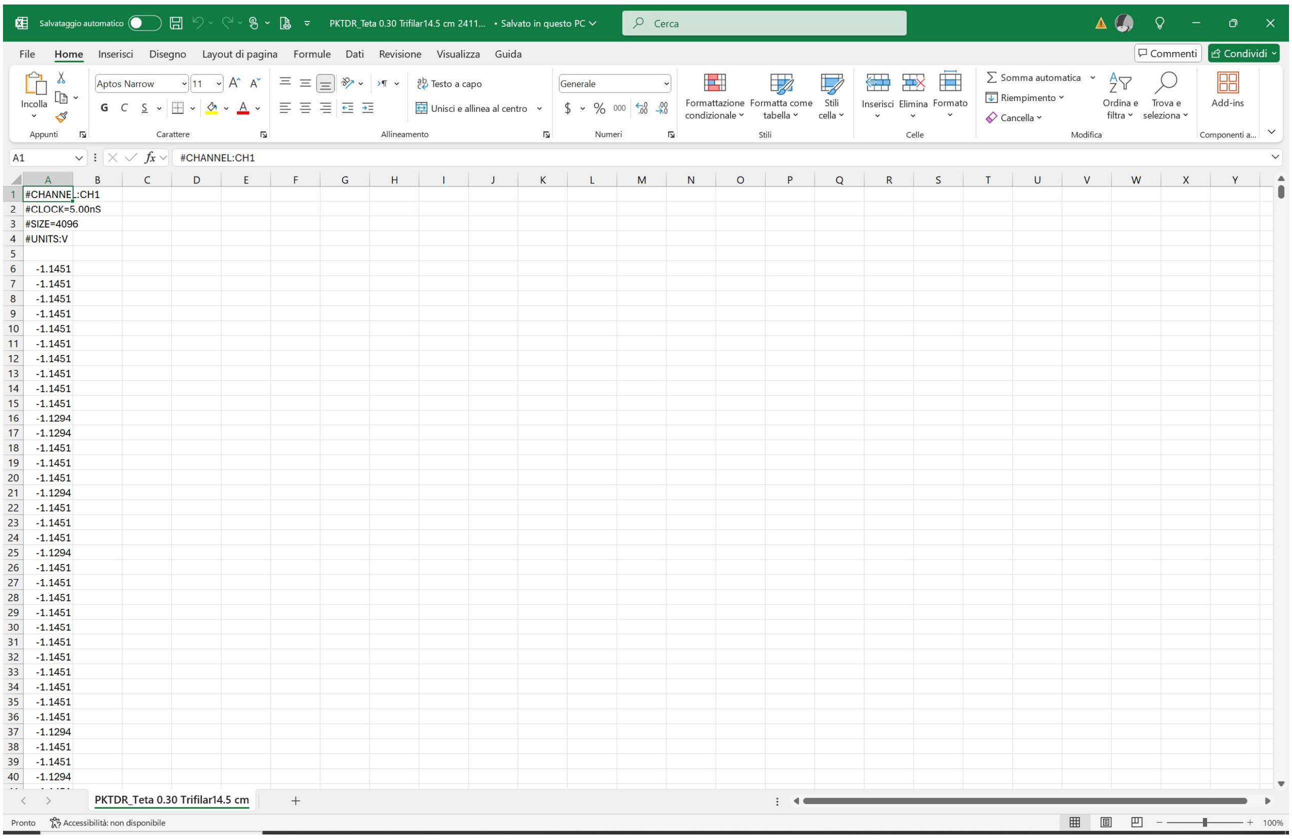

Figure 8 Example of TDR waveform imported into a spreadsheet file.

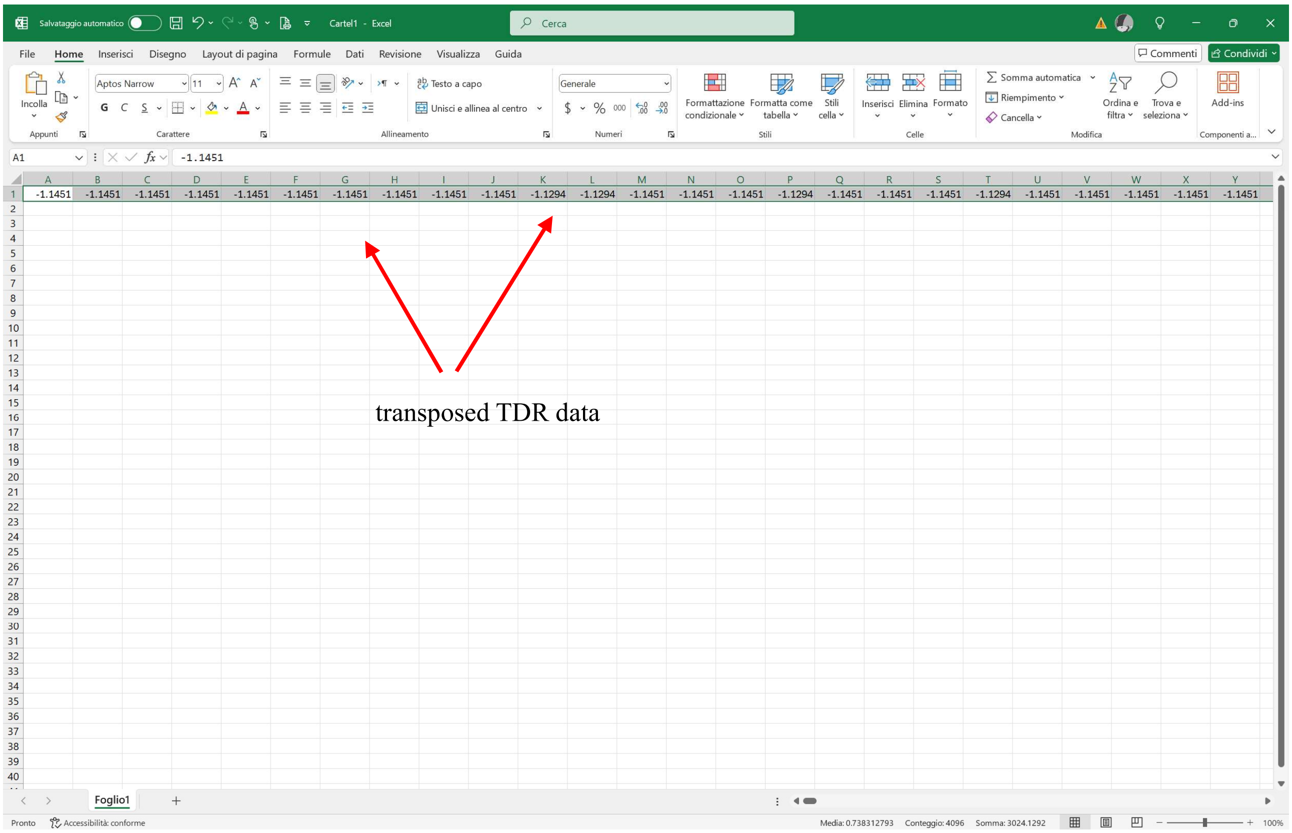

Figure 9 Example transposed TDR waveform.

1.4 The TDROUT.txt output file

As mentioned above, the MATPKTDR code also generates an output text file containing data and time information, along with the estimated bulk dielectric permittivity (epsilon) and volumetric water content (theta). Figure 10 provides an example of this text output file.

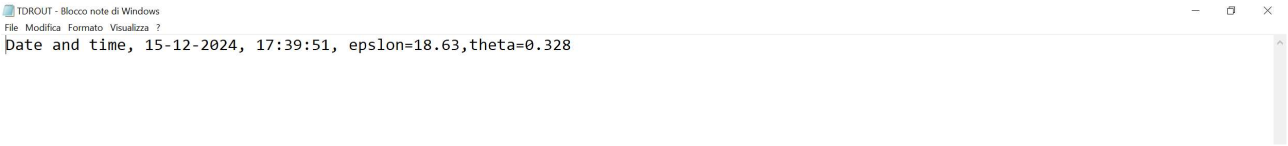

Figure 10 Hard copy of the output generated by the MATPKTDR code.

1.5 The video output

The MATPKTDR code also generates a series of graphical outputs (figure 11). Specifically, the first graphic shows the acquired TDR signal (top left), with the first peak and the calculated reflection point highlighted by red lines. The second graphic is a zoom-in on the first peak-second reflection domain (top right). The third graphic displays the calculated first derivative, which is required for the reflection point calculation (bottom left), and for further analysis, the final graphic shows the calculated second derivative (bottom right).

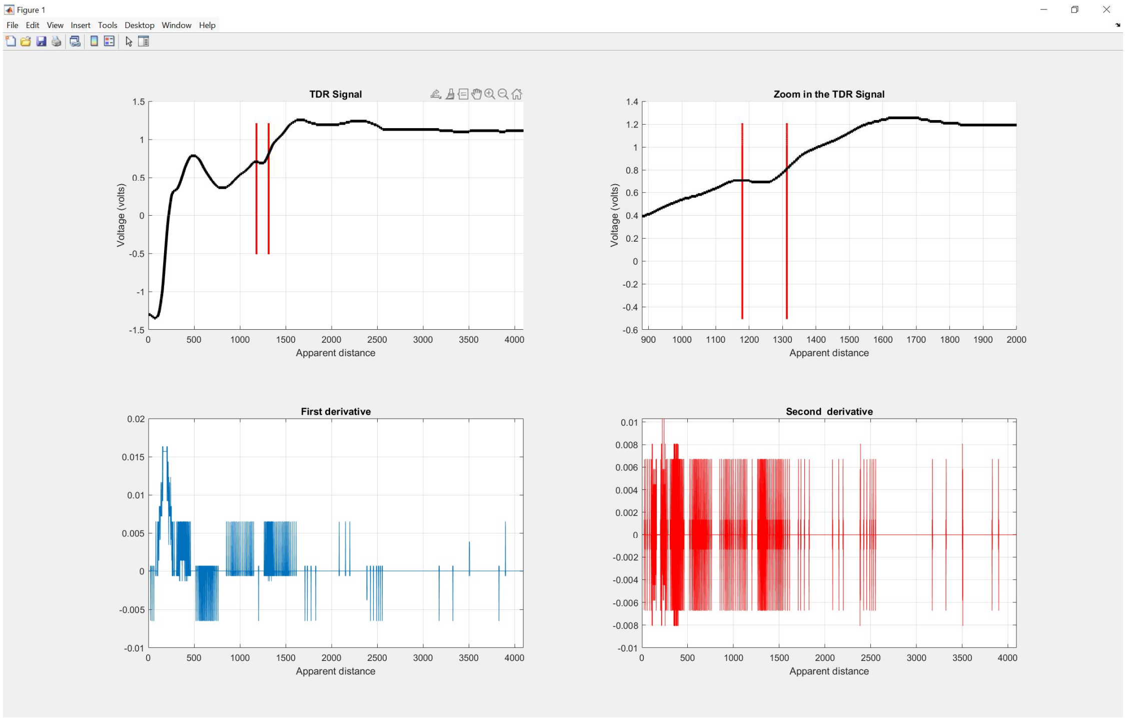

Figure 11 Graphical output produced by the MATPKTDR code for a 4096-point signal.

1.5.1 The video output

The plots of figure 11 are also stored as an external image (**Plots.png**) in the MATPKTDR folder.

2 Example of TDR signal analysis via MATPKTDR

2.1 Processing a TDR signal generated by the PKTDR device connected to the Hantek 6254BD oscilloscope

To process a TDR waveform acquired via PKTDR the user must first prepare the General Trace Information file with the required input parameters. Below is an example of this file.

```
"INPUT FILE DATA: second row: number of points, third row: Time/div, 4th row: Dist/div,
5th row: Div, 6th row: Vp,; 7th row: probe length: 8th row: FirstPeak"
4096
5
0.98
10
0
0.145
1260
```

In our case, we are using a trifilar TDR probe with a length of 14.5 cm (7th row), and the DSO is acquiring a 4096-point signal at a time/division window of 5 ns (3rd row). The DSO display is set to 10 divisions (5th row), and the first peak (determined by the user, as mentioned earlier) is located at a the position 1260. The input file also includes, in the 4th row, information regarding the

Distance/division term (i.e., 0.98), which can be calculated using equation 3. This information is not essential if V_p (6th row) is set to 0.

At this point, the user must create the .csv input file containing the acquired TDR signal, with the TDR values separated by commas. Below is an example of this file.

```
………0.0157,0,0,0.0157,0.0157,0.0314,0.0471,0.0627,0.0784,0.0941,0.0941,0.1098,0.1255,0.1
255,0.1412,0.1569,0.1569,0.1725,0.1882,0.1882,0.2039,0.2039,0.2196,0.2353,0.2353,0.2353
,0.251,0.251,0.2667,0.2667,0.2824,0.2824,0.2824,0.298,0.298,0.3137,0.3137,0.3137,0.3137
,0.3294,0.3294,0.3294,0.3451,0.3451,0.3451,0.3451,0.3608,0.3608,0.3608,0.3608,0.3765,0.
3765,0.3765,0.3765,0.3765,0.3922,0.3922,0.3922,0.3922,0.4078,0.4078,0.4078,0.4078,0.407
8,0.4078,0.4078,0.4078,0.4078,0.4235,0.4235,0.4235,0.4235,0.4235,0.4235,0.4235,0.4235,0
.4235,0.4235,0.4235,0.4235,0.4235,0.4235,0.4235,0.4235,0.4235,0.4392,0.4392,0.4392,0.43
92,0.4392,0.4392,0.4392,0.4392,0.4392,0.4392,0.4392,0.4392,0.4392,0.4549,0.4549,0.4549,
0.4549,0.4549,0.4549,0.4549,0.4549,0.4549,0.4549,0.4549,0.4549,0.4706,0.4706,0.4706,0.4
706,0.4706,0.4706,0.4706,0.4863,0.4863,0.4863,0.4863,0.4863,0.502,0.502,0.502,0.502,0.5
02,0.5176,0.5176,0.5176,0.5176,0.5333,0.5333,0.5333,0.5333,0.5333,0.549,0.549,0.549,0.5
49,0.5647,0.5647,0.5647,0.5647,0.5804,0.5804,0.5804,0.5804,0.5961,0.5961,0.5961,0.5961,
0.8784,0.8784,0.8784,0.8784,0.8627,0.8627,0.8627,0.8627,0.8627,0.8627,0.8627,0.8627,0.8
627,0.8627,0.8627,0.8627,0.8471,0.8471,0.8471,0.8471,0.8471,0.8471,0.8471,0.8471,0.8471
,0.8471,0.8471,0.8471,0.8314,0.8314,0.8314,0.8314,0.8314,0.8314,0.8314,0.8314,0.8314,0.
8314,0.8157,0.8157,0.8157,0.8157,0.8157,0.8157,0.8157,0.8157,0.8,0.8,0.8,0.8,0.8,0.8,0.
8,0.8,0.8,0.8,0.7843,0.7843,0.7843,0.7843,0.7843,0.7843,0.7843,0.7843,0.7843,0.7686,0.7
686,0.7686,0.7686,0.7686,0.7686,0.7686,0.7686,0.7529,0.7529,0.7529,0.7529,0.7529,0.7529
,0.7529,0.7529,0.7373,0.7373,0.7373,0.7373,0.7373,0.7373,0.7373,0.7373,0.7216,0.7216,0.
7216,0.7216,0.7216,0.7216,0.7216,0.7059,0.7059,0.7059,0.7059,0.7059,0.7059,0.7059,0.705
9,0.6902,0.6902,0.6902,0.6902,0.6902,0.6902,0.6745,0.6745,0.6745,0.6745,0.6745,0.6745,0
.6745,0.6588,0.6588,0.6588,0.6588,0.6588,0.6588,0.6588,0.6431,0.6431,0.6431,0.6431,0.64
31,0.6431,0.6431,0.6275,0.6275,0.6275,0.6275,0.6275,0.6275,0.6275,0.6275,0.6118,0.6118,
0.6118,0.6118,0.6118,0.6118,0.6118,0.6118,0.5961,0.5961,0.5961,0.5961,0.5961,0.5961,0.5
961,0.5804,0.5804,0.5804,0.5804,0.5804,0.5804,0.5804,0.5804,0.5804,0.5804,0.5647,0.5647
,0.5647,0.5647,0.5647,0.5647,0.5647,0.5647,0.5647,0.5647,0.549,0.549,0.549,0.549,0.549,
0.549,0.549,0.549,0.549,0.549,0.549,0.5333,0.5333,0.5333,0.5333,0.5333,0.5333,0.5333,0.
5333,0.5333,0.5333,0.5176,0.5176,0.5176,0.5176,0.5176,0.5176,0.5176,0.5176,0.5176,0.517
6,0.5176,0.5176,0.5176,0.5176,0.502,0.502,0.502,0.502,0.502,0.502,0.502,0.502,0.502,0.5
02,0.502,0.502,0.502,0.502,0.4863,0.4863,0.4863,0.4863,0.4863,0.4863,0.4863,0.4863,0.48
63,0.4863,0.4863,0.4863,0.4863,0.4863,0.4863,0.4863,0.4863,0.4706,0.4706,0.4706,0.4706,
0.4706,0.4706,0.4706,0.4706,0.4706,0.4706,0.4706,0.4706,0.4706,0.4706,0.4706,0.4706,0.4
706,0.4706,0.4706,0.4706,0.4706,0.4706,0.4706,0.4706,0.4706,0.4706,0.4549,0.4549,0.4549
,0.4549,0.4549,0.4549,0.4549,0.4549,0.4549,0.4549,0.4549,0.4549,0.4549,0.4549,0.4549,0.
4549,0.4549,0.4549,0.4549,0.4549,0.4549,0.4549,0.4549,0.4549,0.4549,0.4549,0.4549,0.454
9,0.4549,0.4549,0.4549,0.4549,0.4549,0.4549,0.4549,0.4549,0.4549,0.4549,0.4549,0.4549,0
.4549,0.4549,0.4549,0.4549,0.4549,0.4549,0.4549,0.4549,0.4549,0.4549,0.4549,0.4549,0.45
49,0.502,0.502,0.502,0.5176,0.5176,0.5176,0.5176,0.5176,0.5176,0.5176,0.5176,0.5176,0.5
176,0.5176,0.5176,0.5176,0.5176,0.5176,0.5176,0.5176,0.5333,0.5333,0.5333,0.5333,0.5333
,0.5333,0.5333,0.5333,0.5333,0.5333,0.5333,0.5333,0.5333,0.549,0.549,0.549,0.549,0.549,
0., to be continued…
```

Once these files are updated in the main folder (the MATPKTDR folder), the user can launch the MATPKTDR code through the MATLAB Command Window by simply typing: **mainMATPKTDR**. The algorithm will then begin calculations and return the calculated bulk dielectric permittivity (epsilon) and volumetric water content (theta) in the Command Window. Additionally, the code produces a series of graphics (figure 12) and generates a .txt file (TDROUT.txt) containing the obtained results.

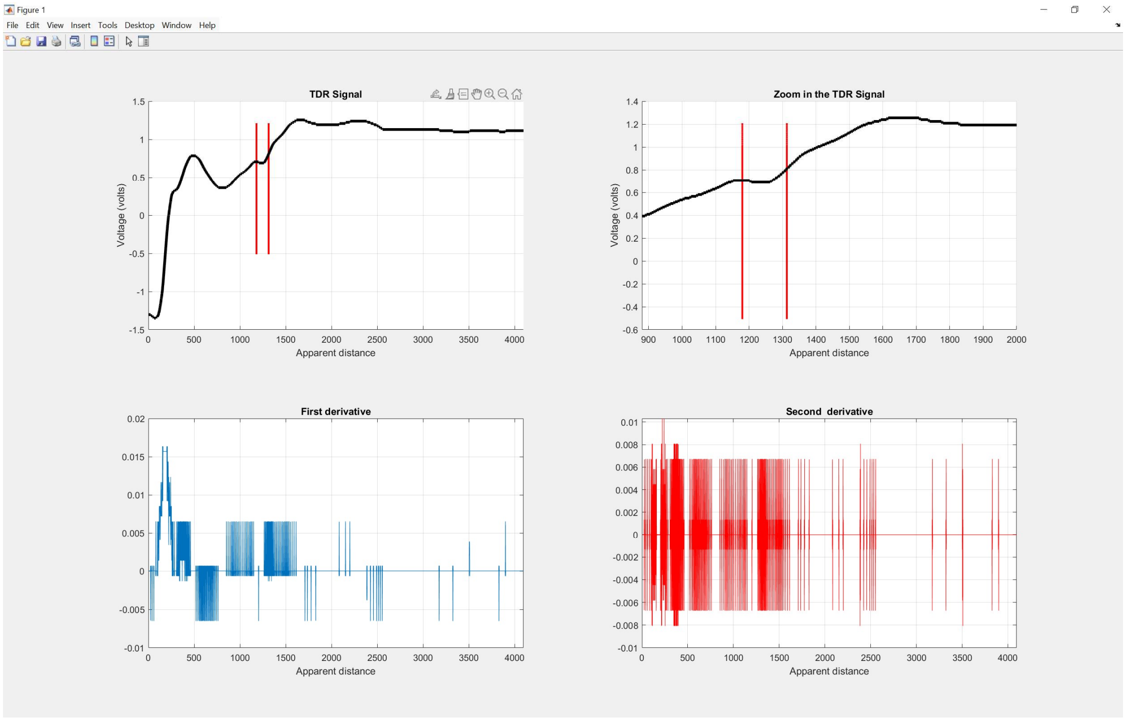

Figure 12 Graphical output produced by the MATPKTDR code for a 4096-point signal.

Below is the output as saved in the TDROUT.txt file.

```
Date and time, 03-12-2024, 10:49:16, epslon=12.63,theta=0.237
```

2.2 2.1 Processing a TDR signal generated by TDR 100 or Tektronix 1502C

In this case, the TDR waveform is acquired using the TDR100 or Tektronix 1502C. The user must first prepare the General Trace Information file with the required input parameters. Below is an example of this file.

```
"INPUT FILE DATA: second row: number of points, third row: Time/div, 4th row: Dist/div, 5th row: Div, 6th row: Vp,; 7th row: probe length: 8th row: FirstPeak"
251
0
0.25
10
0.66
0.145
19
```

At this stage, the user must create the .csv input file containing the acquired TDR signal, with TDR values separated by commas. Below is an example of this file.

```
0,0.0577,0.0587,0.0515,0.0525,0.0473,0.0556,0.0567,0.0577,0.0525,0.0577,0.066,0.0556,0.
066,0.0671,0.092,0.1625,0.2051,0.1656,0.1189,0.0857,0.0525,0.0328,0.0058,-0.0139,-
0.0129,-0.0274,-0.0471,-0.071,-0.0668,-0.0845,-0.0886,-0.1073,-0.1052,-0.0689,-
0.0004,0.0899,0.1522,0.2113,0.2476,0.2674,0.2943,0.3109,0.3244,0.3141,0.3296,0.3348,0.3
4,0.3452,0.3545,0.3659,0.3774,0.3857,0.3929,0.4147,0.4241,0.4209,0.4158,0.4106,0.4095,0
.4033,0.4126,0.4178,0.4189,0.4189,0.4137,0.4116,0.4261,0.4209,0.4168,0.422,0.4251,0.426
1,0.4251,0.423,0.4158,0.422,0.422,0.4199,0.4241,0.4313,0.4313,0.4313,0.45,0.45,0.4531,0
.4521,0.4604,0.4479,0.4542,0.4573,0.4459,0.4438,0.451,0.4604,0.4614,0.4614,0.4562,0.460
4,0.4542,0.451,0.4479,0.451,0.449,0.4604,0.4583,0.4552,0.4604,0.4687,0.4687,0.4645,0.47
```

```
39,0.4697,0.4677,0.4697,0.4739,0.4749,0.476,0.4697,0.4791,0.4739,0.477,0.4801,0.4604,0.
4728,0.476,0.4728,0.4697,0.4625,0.4645,0.4677,0.4791,0.4728,0.4687,0.4791,0.4749,0.4708
,0.4728,0.4811,0.476,0.477,0.4822,0.4832,0.4863,0.4822,0.4832,0.4843,0.4843,0.4843,0.48
63,0.4863,0.4811,0.4853,0.4843,0.4801,0.4811,0.4822,0.4832,0.4791,0.4863,0.4853,0.4884,
0.4936,0.4832,0.4791,0.477,0.4708,0.4843,0.4843,0.4801,0.478,0.4739,0.4884,0.4811,0.478
,0.4749,0.477,0.4749,0.4791,0.4811,0.4832,0.478,0.4832,0.4853,0.4884,0.4884,0.478,0.481
1,0.4791,0.4728,0.4863,0.4884,0.4884,0.477,0.4811,0.4801,0.4853,0.4843,0.4811,0.478,0.4
863,0.4863,0.4863,0.4884,0.4915,0.4957,0.4926,0.4988,0.4915,0.4811,0.4801,0.4791,0.4863
,0.4832,0.4822,0.4843,0.4822,0.4843,0.4801,0.4832,0.4843,0.4822,0.4874,0.4822,0.4905,0.
4957,0.4905,0.4811,0.4936,0.4884,0.4926,0.478,0.4884,0.4915,0.4905,0.4884,0.4905,0.476,
0.4791,0.4801,0.4936,0.4863,0.4926,0.4936,0.4926,0.4811,0.4967,0.4926,0.4791,0.476,0.48
84
```

The TDR probe is trifilar with a length of 14.5 cm (7th row), and the TDR system is acquiring a 251-point signal at a Distance/division window of 0.25 (3rd row). The second line is fixed to 0, as these commercial devices operate in an apparent distance domain. The TDR device is set to 10 divisions (5th row), and the first peak (determined by the user, as mentioned above) is located at a bulk distance of 19. The V_p term (6th row) is set to 0.66.

Once the input file is updated, the user can launch the MATPKTDR code through the MATLAB Command window by simply typing **mainMATPKTDR**. The algorithm will then begin calculations and return the calculated bulk dielectric permittivity (epsilon) and volumetric water content (theta) in the Command Window. Figure 13 shows the video output.

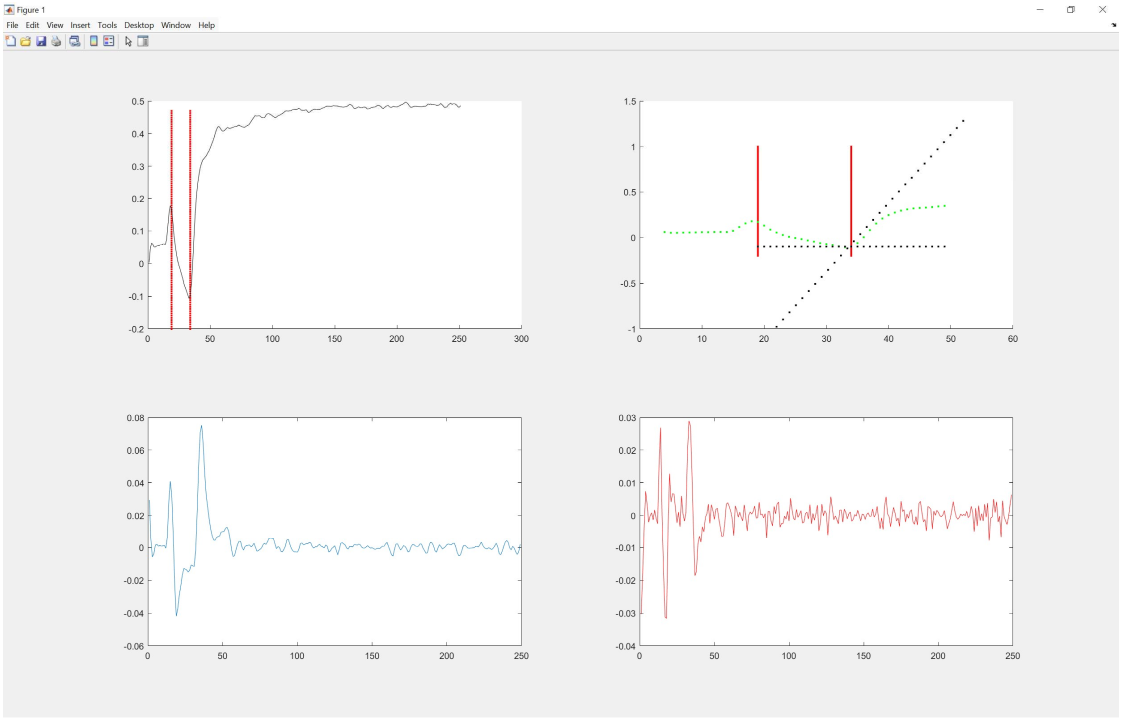

Figure 13 Graphical output produced by the MATPKTDR code for a 251-point TDR signal.

Below is the output as saved in the TDROUT.txt file.

```
Date and time, 03-12-2024, 12:42:49, epslon=13.40,theta=0.250
```

References

Topp, G.C., J.L. Davis, and A.P. Annan. 1980. Electromagnetic determination of soil water content: Measurement in coaxial transmission lines. Water Resour. Res. 16:574–582. doi:10.1029/WR016i003p00574.

Appendix: How to assemble the PKTDR system

The PKTDR system requires additional components to function correctly. In addition to a DSO, the system needs a resistive load (PK57 model) to ensure a stable 50-ohm output signal. A three-way BNC connector is also necessary to facilitate connections between the PKTDR, the TDR probe, and the DSO. Finally, both the DSO and the PKTDR need to be powered (at 5 volts) and connected to a PC. For this, USB-C cables are required. Figure A1 shows a scheme of the required components and connections.

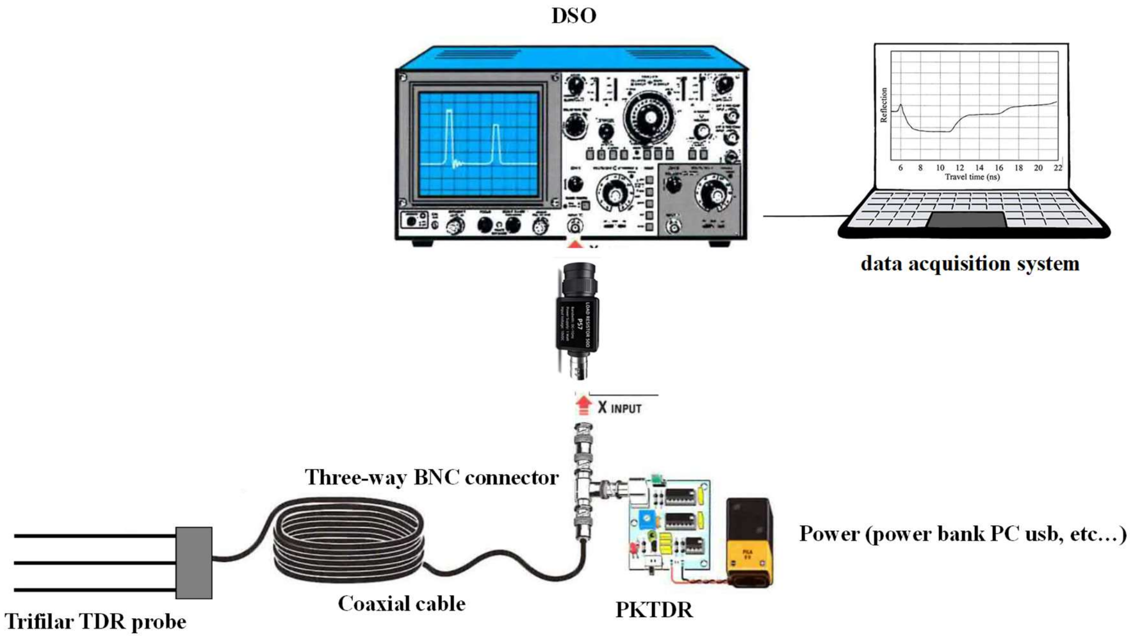

Figure A1 Scheme of the required components and connection for the PKTDR system.

More specifically, the PKTDR and the TDR probe must be connected to the DSO via the three-way BNC connector. Between this connection and the DSO, the P57 load resistor should be placed. The DSO needs to be connected to the PC via a USB cable, and both the PC and the PKTDR must be powered (either together, for example through the PC, or separately).
