# Supplementary figures and images for: A Novel Time Domain Reflectometry (TDR) System for Water Content Estimation in Soils: Development and Application
Source: Sensors (Basel). 2025 Feb 12;25(4):1099. doi: 10.3390/s25041099 (PMC11859294; doi:10.3390/s25041099)

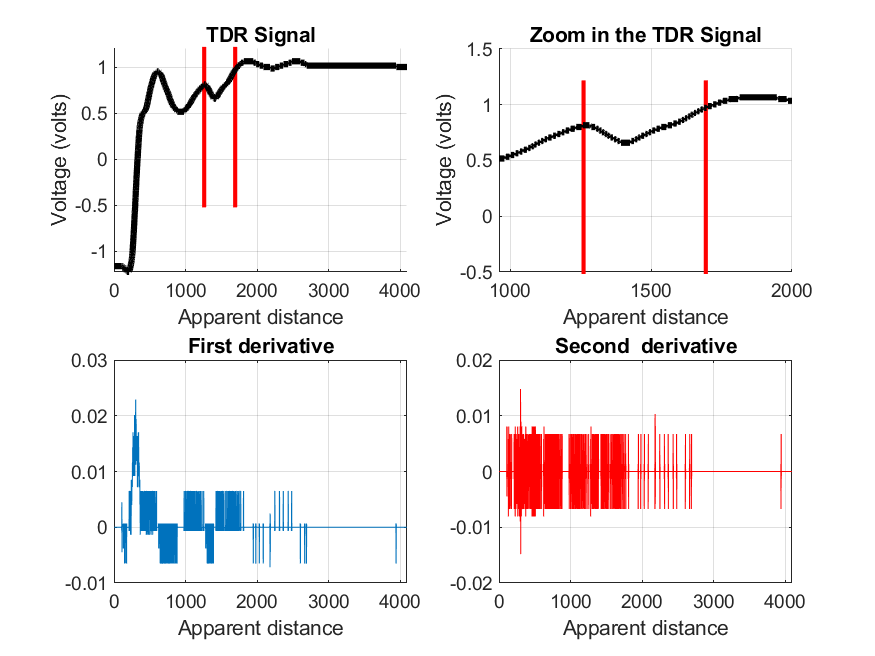

Supplement: Supplementary file 1 [file sensors-25-01099-s001.zip › 02 MATPKTDR Software folder/MATPKTDR/Plots.png]
